# Acknowledgement to Reviewers of *Pharmaceutics* in 2014

**DOI:** 10.3390/pharmaceutics7010001

**Published:** 2015-01-08

**Authors:** Pharmaceutics Editorial Office

**Affiliations:** MDPI AG, Klybeckstrasse 64, CH-4057 Basel, Switzerland

The editors of *Pharmaceutics* would like to express their sincere gratitude to the following reviewers for assessing manuscripts in 2014:

Acosta, Edgar
Agu, Remigius
Alexopoulos, Aleck H
Anadon, Arturo
Antipina, Maria N.
Azmi, Asfar
Babu, R. Jayachandra
Beck-Broichsitter, Moritz
Broutin, Sophie
Burley, Jonathan C.
Caramella, Carla
Cevc, Gregor
Chatel, Jean-Marc
Cheung, Chi Wai
Chiang, Po-Chang
Chiu, Loraine LY
Choy, Jin-Ho
Clark, Meredith
Costantino, Luca
Costantino, Umberto
D’souza, Martin J.
Das, Diganta B.
Donnelly, Ryan F.
Douglas, Timothy
Douroumis, Dionysios
Ehrhardt, Carsten
Ensign, Laura
Erxleben, Andrea
Fang, Xie
Fink, James B.
Frasch, H. Frederick
Fresta, Massimo
Gadkar, Kapil
Garbacz, Grzegorz
Ghanotakis, Demetrios F
Ham, Anthony S.
Hanada, Sanshiro
Hedoux, Alain
Hirayama, Fumitoshi
Jacobsen, Jette
Javier Gonzalez, Francisco
Kalia, Yogeshvar N.
Kan, Ch-Wai
Kang, Lifeng
Kawakami, Kohsaku
Kennedy, David W
Knott, R.
Kulozik, Ulrich
Lam, Kim-Hung
Lee, Chia-Hung
Leporatti, Stefano
Lin, Paulo J.C.
Liu, Fang
Longest, P. Worth
Lu, Jun
Maggio, Edward T.
Mansour, Heidi M.
Martino, Piera Di
Mathias, Neil R.
McConville, Christopher
Milanesio, Marco
Narjoz, Celine
Neves, J. das
O’Leary, Stephen
Oh, Jae-Min
Oyewumi, Moses
Papineni, Arathi
Paquette, Benoit
Paradossi, Gaio
Park, Ji-Young
Pea, Federico
Perissutti, Beatrice
Petroni, Debora
Rades, Thomas
Ribeiro, Ana C.f.
Rodrigues, Alírio E.
Rohan, Lisa
Rosania, Gus
Sahajwalla, Chandrahas G.
Salcedo, Inmaculada
Salmaso, Stefano
Saluja, Ashok
Sarmento, Bruno
Shahzad, Yasser
Sparen, Anders
Stewart, Clinton F.
Su, Nan-Wei
Tang, De-Chu
Tho, Ingunn
Tian, Furong
van den Berg, Jacob J.
Vanić, Zeljka
Walzel, Peter
Watts, Peter
Yeo, Yoon
Zhou, Yue
Zumbuehl, Andreas

